# Protocol for project IMPACT (improving millions hearts for provider and community transformation): a quasi-experimental evaluation of an integrated electronic health record and community health worker intervention study to improve hypertension management among South Asian patients

**DOI:** 10.1186/s12913-017-2767-1

**Published:** 2017-12-06

**Authors:** Priscilla M. Lopez, Jennifer Zanowiak, Keith Goldfeld, Katarzyna Wyka, Ahmad Masoud, Susan Beane, Rashi Kumar, Phoebe Laughlin, Chau Trinh-Shevrin, Lorna Thorpe, Nadia Islam

**Affiliations:** 1 Department of Population Health, NYU School of Medicine, New York, USA; 2 NYU-CUNY Prevention Research Center, New York, USA; 3 CUNY Graduate School of Public Health and Health Policy, New York, USA; 4 Consulting Firm: iRCM, Inc., New York, USA; 5 Healthfirst, New York, USA

**Keywords:** Community health workers (CHWs), Health information technology (HIT), Electronic health record (EHR), Community-clinical linkages, Hypertension, Million hearts® initiative, Immigrant health, South Asians

## Abstract

**Background:**

The Million Hearts® initiative aims to prevent heart disease and stroke in the United States by mobilizing public and private sectors around a core set of objectives, with particular attention on improving blood pressure control. South Asians in particular have disproportionately high rates of hypertension and face numerous cultural, linguistic, and social barriers to accessing healthcare. Interventions utilizing Health information technology (HIT) and community health worker (CHW)-led patient coaching have each been demonstrated to be effective at advancing Million Hearts® goals, yet few studies have investigated the potential impact of integrating these strategies into a clinical-community linkage initiative. Building upon this initiative, we present the protocol and preliminary results of a research study, Project IMPACT, designed to fill this gap in knowledge.

**Methods:**

Project IMPACT is a stepped wedge quasi-experimental study designed to test the feasibility, adoption, and impact of integrating CHW-led health coaching with electronic health record (EHR)-based interventions to improve hypertension control among South Asian patients in New York City primary care practices. EHR intervention components include the training and implementation of hypertension-specific registry reports, alerts, and order sets. Fidelity to the EHR intervention is assessed by collecting the type, frequency, and utilization of intervention components for each practice. CHW intervention components consist of health coaching sessions on hypertension and related risk factors for uncontrolled hypertensive patients. The outcome, hypertension control (<140 mmHg systolic blood pressure (BP) and <90 mmHg diastolic BP), is collected at the aggregate- and individual-level for all 16 clinical practices enrolled.

**Discussion:**

Project IMPACT builds upon the evidence base of the effectiveness of CHW and Million Hearts® initiatives and proposes a unique integration of provider-based EHR and community-based CHW interventions. The project informs the effectiveness of these interventions in team-based care approaches, thereby, helping to develop relevant sustainability strategies for improving hypertension control among targeted racial/ethnic minority populations at small primary care practices.

**Trial registration:**

This study protocol has been approved and is made available on Clinicaltrials.gov by NCT03159533 as of May 17, 2017.

## Background

The Million Hearts® initiative aims to prevent heart disease and stroke in the United States (US) by mobilizing public and private sectors around a core set of objectives, with particular attention on improving blood pressure control [1]. Nationwide, there are 67 million adults with hypertension, accounting for nearly a third (30.3%) of the population. Fewer than half of these individuals have achieved control despite the wide availability of affordable generic medications and their effectiveness in reducing all-cause hospitalization risk and total health care costs [2, 3]. The burden of hypertension is particularly high in minority groups, including certain Asian American subgroups.

South Asians have disproportionately high rates of cardiovascular disease (CVD), with a unique profile of associated risk factors, including high rates of hypertension and diabetes, low rates of hypercholesterolemia and a different distribution of obesity across CVD risk factor groups [4–17]. Age-adjusted prevalence of hypertension (diagnosed and undiagnosed together) among South Asians in New York City (NYC) is estimated to be 43%, compared to 27.5% among white adults, according to results from the 2013/2014 NYC Health and Nutrition Examination Survey (NYC HANES) [18]. South Asians face numerous cultural, linguistic, and social barriers to accessing healthcare, which may impede clinical and self-management of hypertension [19-25]. Providers may also lack the appropriate tools and resources to identify and manage South Asian hypertensive patients or refer them to culturally appropriate programs that support hypertension and diabetes control. Programs already proven effective in other communities can be adapted to address this gap and effectively improve hypertension management in the South Asian population.

Individually, health information technology (HIT) and community health worker (CHW)-led patient coaching interventions have each demonstrated effectiveness at advancing Million Hearts® goals, yet few studies have investigated the potential impact of integrating these strategies into a clinical-community linkage initiative [26–31]. Data available through electronic health records (EHRs) can be used to identify candidates for needed follow-up, targeted risk-reducing interventions and can be designed to allow primary care providers (PCPs) to easily refer patients to counseling and other services [32–35]. Adding CHWs to the primary care team can improve care for patients with chronic disease(s) at low cost [36]. Studies are now beginning to demonstrate that EHR access and communication between the PCP and CHW can facilitate the acceptance and effectiveness of emerging care management models and lead to improved patient outcomes [37, 38].

Building upon the strategies of the Millions Hearts® initiative and the evidence base of the effectiveness of CHW interventions, we present the protocol of a research study designed to understand the effectiveness of integrating these approaches towards hypertension management among a South Asian population [1]. Project IMPACT is a 5-year stepped wedge quasi-experimental study designed to test the feasibility, adoption, and impact of integrating a CHW-led health coaching with practice- and provider-level EHR-based interventions to improve hypertension control among South Asian patients in NYC primary care practices.

## Methods

### Objectives

The primary aim of the current study is to assess the effectiveness of an integrated EHR and CHW intervention to improve hypertension control among South Asian patients with poorly controlled hypertension in 16 NYC primary care practices.

### Study team

This study is led by researchers from the New York University-City University of New York Prevention Research Center (NYU-CUNY PRC), funded by the Centers for Disease Control and Prevention [39]. The NYU-CUNY PRC is a public-private academic partnership between the NYU School of Medicine and the CUNY School of Public Health, and its mission is to implement, evaluate, and disseminate community-clinical linkage interventions to reduce cardiovascular disease disparities in ethnically diverse NYC communities. For this project, researchers from the NYU-CUNY PRC partnered with Healthfirst (HF), a not-for-profit managed care organization serving more than 35,000 South Asian members in NYC. Primary care practices enrolled into the study are part of HF’s provider network [40]. The team also engaged with IPRO, the federally-funded Medicare Quality Innovation Network-Quality Improvement Organization for New York State, Washington D.C. and South Carolina, under contract with the Centers for Medicare & Medicaid Services (CMS), to train provider networks on the use of EHR systems in order to implement and monitor Million Hearts® goals, as well as training on consistency in blood pressure recordings [41]. A coalition of South Asian community-based organizations with expertise in the development and implementation of culturally tailored community-clinical linkage models was engaged to provide feedback on the CHW component of the intervention, including reviewing and adapting CHW curriculum and patients materials [42, 43]. An independent EHR consultant was also hired to provide technical assistance to practices throughout the course of the intervention.

### Ethics and data sharing approvals

All study protocol and procedures were reviewed by the Institutional Review Board at the NYU School of Medicine and the CUNY School of Public Health. Primary care practices identified as potential intervention sites signed two Memorandums of Understanding (MOUs) between the primary care practice and study team for each component of the intervention. For the EHR component of the intervention, the MOU included the following components: (1) EHR intervention components to be implemented; (2) training requirements; (3) aggregate and individual-level patient data extraction, confidentiality and storage procedures associated with the study. For the CHW component of the intervention, the MOU detailed: (1) recruitment strategies; (2) CHW intervention components to be implemented; and (3) individual-level patient data extraction, confidentiality and storage procedures associated with the study. Written informed consent is obtained from study participants receiving CHW services. This component of the intervention was registered via ClinicalTrials.gov by NCT03159533 as of May 17, 2017. Finally, participating academic institutions signed a data use agreement with HF that provided guidelines and protections around the usage and storage of data for research purposes.

### Study design

We use a stepped wedge design to simultaneously analyze the effectiveness and implementation process of a multi-component quality improvement intervention on hypertension control tailored for a unique minority population [44, 45]. The intervention is implemented in two phases in 16 primary care practices in NYC that are part of the HF network and serve large numbers of South Asian patients. The first year of the study is dedicated to recruitment of sites, planning, and determining the specific components of the EHR and CHW interventions to be implemented. In Years 2 to 3, we implement and evaluate the EHR phase of the intervention within 16 PCP practice sites. In Years 3 to 4, we integrate the CHW-led coaching and patient education phase of the intervention with the EHR physician-level efforts in all sites. In Year 5, we assess the implementation process and use findings to develop a set of best practices and toolkits for public health and healthcare agencies regarding integrated EHR-CHW strategies to improve hypertension control.

**Fig. 1:**
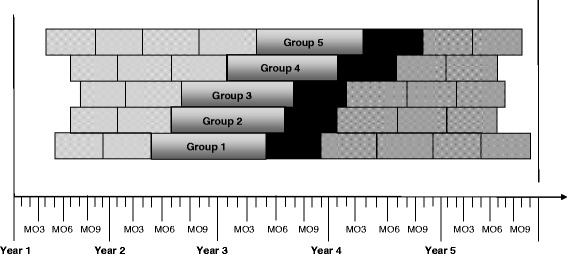
Modified Stepped Wedge Design for Project IMPACT. Key:  = Baseline Period (6-month intervals),  = Adoption of EHR Intervention,  = Adoption of CHW Intervention,  = Follow-up Data (6-month intervals)

The study design employs staggering of the EHR and integrated EHR-CHW interventions using a modified stepped wedge design. The modifications to the traditional stepped wedge design include lack of random allocation of the intervention into the practice sites due to logistical and acceptability concerns on the part of practices, and variations in the length of time between steps [44, 45]. Figure 1 demonstrates the stepped wedge design, where groups 1–5 represent clusters of 2–4 practices that have been progressively allocated into the components of the intervention by enrollment date. Data related to the study outcomes are extracted from EHR systems on a biannual basis. By the beginning of Year 3, the EHR intervention is implemented at all sites, and by the end of year 3, the linked CHW intervention is implemented at all sites [37, 38, 43].

### Intervention components

#### Phase 1: EHR intervention

The EHR intervention components of the project were developed with input from project partners and participating clinics, and are centered on 1) generating routine hypertension patient registry reports within each practice; and 2) developing and implementing medical alerts and order sets tailored to the South Asian patient population. The features of the EHR intervention component are implemented at the participating sites after careful considerations to their baseline workflow, staff capacity, and logistical feasibility conducted through a mixed methods baseline assessment. Recommendations are made for a revised practice workflow to ensure that the proposed EHR intervention component is practical, realistic, and tailored for each individual practice.

*Patient Registry -* The patient registry is a feature within the EHR that allows providers to query and group information on their patients based on specified criteria, ranging from diagnoses of particular health conditions to demographic characteristics [46]. The registry allows practices to plan and prioritize patient visits, identify potential care required, and measure overall practice performance. This project provides the clinical team with training on the following competencies: understanding the functionality and potential impact of the registry; appropriately generating registry reports for follow-up care; to identify patients that have been lost-to-follow up; to incorporate registries into the day-to-day office activities; and to monitor the use, satisfaction and impact of utilizing the patient registry over time. Specifically, the project focuses on encouraging the routine generation of registry reports that identify patients with diagnosed hypertension that had a poorly controlled blood pressure reading at last clinic visit in order to help providers prioritize a follow-up visit from these patients. *Alerts –* Alerts may be built within the EHR to remind staff and providers to complete a particular action at the point of care [47]. These alerts can be patient-specific or “global,” meaning that they apply to any patient that satisfies specified criteria. This project provides the clinical team with training on understanding functionality and potential impact of these alerts and how to utilize them for the prevention and management of hypertension. Alerts are tailored to trigger when a blood pressure measurement is missing for the patient at the point of care, and prompts the provider for a repeat measure if elevated. In addition, for patients with a diagnosis of hypertension, an alert prompts the clinical team to create an appointment for the patient to return to the practice within 6 months if blood pressure is controlled, or within 1 month if poorly controlled [48]. *Order sets –* Order sets are standardized sets of evidence-based treatment guidelines that apply when linked to an alert [47, 48]. In this project, we create an order set that includes a combination of prescriptions, lab tests, and counseling orders “pre-set” for patients with hypertension. Within the counseling orders, we upload evidence-based, culturally tailored, in-language educational materials for distribution to patients fluent in a variety of South Asian languages.

To complement these components, participating practice staff are trained on creating customizable templates that increase the efficiency and accuracy with which staff documents vital signs and other pertinent health data, and utilizing automated appointment reminder texts and letters that can be sent to patients electronically [47]. In tandem, practices are encouraged to collaborate with IPRO, a partner in this intervention, to participate in EHR-based incentive programs, such as Meaningful Use (MU) and National Committee for Quality Assurance (NCQA) Patient-Centered Medical Home (PCMH) recognition [49, 50].

#### Phase 2: CHW intervention

One year after each participating clinical practice implements the EHR intervention, the CHW intervention launches. To prioritize eligible patients to enroll in this intervention, a list of South Asian patients with > = 140 systolic blood pressure and/or > = 90 diastolic blood pressure at last visit within the last 6 months is generated from the EHR at each of the participating PCP sites. Eligible patients receive a letter from their physicians inviting them to participate in the CHW intervention (a series of group and one-on-one educational sessions on hypertension self-management), and CHWs follow-up with a telephone call. CHWs were trained in a set of core competencies, including cardiovascular disease, mental health, motivational interviewing, smoking cessation, and other related topics.

CHWs deliver a standardized curriculum on hypertension management adapted from the National Heart, Lung, and Blood Institute (NHLBI) Healthy Heart, Healthy Family program, other community-based CHW and heart health educational curriculum implemented in South Asian communities, and relevant Million Hearts® Initiative materials [1, 42, 43, 51, 52]. The protocol consists of 5 monthly 60-min group health education sessions that provide the tools and strategies to promote heart health to South Asian individuals, families, and communities on high blood pressure and CVD [1, 42, 43]. All sessions employ adults learning techniques and group-based learning and activities, and materials have been culturally and linguistically adapted. Each of the group health education sessions discuss different content related to CVD, hypertension, and other CVD risk factors that are culturally relevant for South Asian populations (Table 1).

**Table 1:** Community Health Worker (CHW) Intervention Curriculum

Session Topic	Session Overview	Tailored Cultural Components
Session 1: Blood Pressure and the Cardiovascular System	*Icebreaker/Introduction and Session Guidelines*	• Highlight local health and social services resources, as well as risk factors for South Asians
	1.How the heart works & heart structure	
	2. What is blood pressure and hypertension (BP numbers)	
	3. How to check your blood pressure (demo and practice)	
	4. Risk factors of hypertension	
	5. Ways to manage blood pressure: healthy diet, physical activity, medicine (overview)	
	6. Signs of heart attack and stroke & Emergency Plan	
	7. Physical Activity Exercises (demo and practice)	
Session 2: Healthy eating	1.Traditional South Asian diets (discussion)	• Food examples tailored for South Asian diets and dietary practices
	2. Building a healthy plate (Using Plate Method)	
	3. How to choose heart healthy foods	
	4. Salt and sodium	
	5. How to understand a nutrition label	
	6. Alcohol	
	7. Tips for healthy eating while out, with little time, and on a budget	
	8. Setting healthy eating goals	
Session 3: Physical Activity and Stress Management	1. Importance of physical activity	• Use of Asian BMI guidelines
	2. What is a healthy weight/BMI?	• Realistic exercise options in NYC communities
	3. Calorie balance and the healthy way to lose weight	• Discussions on major stressors and ways to reduce stresses in South Asian context
	4. Ways to be active, build activity into your day, and stay motivated	
	5. Sample exercises and walking program	
	6. Setting physical activity goals	
	7. Effect of stress on the body	
	8. Emotions like anger, frustration, sadness, worry	
	9. Strategies to manage stress improve self-esteem	
Session 4: CVD risk factors: cholesterol, blood sugar, & smoking	1. Facts about saturated fat, trans fat, and cholesterol	• Discussion of CVD risk factors, including smoking and tobacco use, is contextualized into South Asian context
	2. Understanding nutrition labels	
	3. Healthier cooking tips	
	4. Diabetes - What is it, types, and symptoms	
	5. Complications of diabetes & diabetes control	
	6. Hidden sugar in drinks activity	
	7. Effect of smoking and tobacco use on health	
Session 5: Health Communication, Healthcare access & sessions review	1. Communicating with doctors	• Discussion of barriers to healthcare for South Asian patients
	2. Barriers to healthcare access	
	3. Preparing for a doctor visit	
	4. Accessing health care	
	5. Review of all sessions	

Sessions are held in PCP offices and other community spaces, and multiple timeslots of each session are held to accommodate patients’ varying schedules. Between sessions, CHWs follow up with participants at least bi-weekly by phone or in-person through a home or clinic visit. At these sessions or calls, CHWs engage in goal-setting activities regarding changes to health behaviors, medication adherence, or other issues related to hypertension control as identified jointly by patient and CHW. The CHW also makes necessary referrals to other services available in the community (i.e. exercise classes, social services, mental health, tobacco cessation, etc.).

Recruitment and eligibility.

### Practices

Working in concert with HF, we identified independent PCPs in Queens and Brooklyn with multiple or single PCPs at each site that are part of HF’s network and serve significant numbers of South Asian patients (defined as practices with at least 100 HF patients, and more than 70% of patients identifying as South Asian, or over 100 South Asian patients with hypertension). Practices were required to have an operating EHR, specifically eClinicalWorks (eCW) or MDLand, for at least 12 months prior the time of the enrollment [53, 54]. These sites were contacted by HF or the NYU-CUNY PRC study team staff by telephone to assess their eligibility in terms of number of South Asian patients and general interest in the project. If the representative expressed interest, the study team scheduled a site visit, during which eligible and interested practices signed an MOU to participate in the EHR intervention, and, one year later, a second MOU to participate in the CHW intervention.

### Participants

Prior to the launch of the CHW intervention at each site, NYU-CUNY PRC staff work in concert with practice staff to identify a list of hypertensive patients within the previous six months that, at the last office visit, were reported to have a blood pressure > = 140 systolic or > = 90 diastolic through the EHR health registry. These patients are contacted by the CHWs and encouraged to participate if eligible. To be eligible, these patients must be between 18 and 85 years of age and must not be pregnant at the time of screening. If eligible, patients complete an in-person or phone-based screening that assesses baseline demographic and logistical information, such as preferred language and availability for education sessions. CHWs also participate in ‘tabling’ at practices, where CHWs ask interested patients in the waiting room to complete a screening form. For patients not identified through the original list, but found to have an uncontrolled blood pressure reading during the screening, they are encouraged to participate. To enroll into the intervention, patients must sign a consent form. All eligible and consented patients are then randomized, within each site, to participate in the CHW intervention either immediately or in six months. The purpose of this randomization is to have the second group, participating 6 months later, serve as a comparison group to the first group, participating immediately.

#### Data collection, measures, primary study outcomes, and analysis

The intervention launched in January 2016 at the first round of clinical practices, with additional rounds recruited at ~3-month intervals across the next year. The baseline period for each site is defined as the period prior to launch of the EHR intervention measured in 6-month intervals that occurred after Dec 31, 2015. Thus, all practices have a minimum of two 6-month baseline periods, and some have up to 4 rounds. Each practice is considered to be in the intervention period after the 3rd day of the EHR intervention launch, and a group intervention start date is selected for each round of clinical practices to accord with the first day of the next month. The intervention period includes the group start date through August 1, 2019.

### Primary study outcome at practice level: Aggregate BP

Aggregate, or practice-level, EHR data is extracted for each 6-month time period between start of baseline and the start of the CHW intervention to evaluate the effect of the EHR intervention components. This data is also collected from the launch of the CHW intervention to August 2019 to evaluate the integrated effect of the EHR and CHW interventions. The primary outcome is the proportion of patients with a diagnosis of hypertension seen in the past 6 months deemed to be well-controlled (systolic blood pressure < 140 and diastolic blood pressure < 90) at last visit, following the indicator definition recommended by the NCQA [51]. The denominator includes all patients diagnosed with hypertension. Data extracted also includes demographics for patients seen for an office visit, as well as the proportion of these patients with a diagnosis of hypertension in the EHR by demographic subgroup (age group, sex). In addition, we monitor the proportion of hypertensive patients that report being a smoker and the proportion of these patients who were referred to smoking cessation during the past 6 months.

The impact of the EHR intervention on proportion of patients seen in the past 6 months with a diagnosis of hypertension deemed to be well-controlled at last visit will be examined in a mixed effects Poisson model, using pre-intervention time periods for comparison (see eq. 1):

1 $$ \log \left({C}_{it}\right)=\mu +{\beta}_1t+{\beta}_2{I}_{it}+{\beta}_3{I}_{it}\left(t-{s}_i\right)+{b}_i+\log \left({E}_{it}\right), $$

where *C*_*it*_ is the number of patients in site *i* who had controlled hypertension during period *t*. Each period is 6 months, and *t* = 0 is the baseline period. *I*_*it*_ is an indicator variable, and *I*_*it*_ = 1 if site *i* has started using the EHR at period *t*, *I*_*it*_ = 0 otherwise. *s*_*i*_ is the time period when the EHR starts at site *i*. *E*_*it*_ is the number of patients at site *i* who were diagnosed as hypertensive during period *t*. Log(*E*_*it*_) is considered the “offset” in the Poisson regression model. *b*_*i*_ is a random effect for site *i* with mean 0 and variance ${\upsigma}_b^2$. The estimation via model (1) takes into account a general time trend, and allows for the intervention effects to grow over time following implementation of intervention. Our primary outcome is the proportion of patients who have controlled hypertension after the site has been using the EHR intervention components in the previous 6 months. This effect (after taking into account general time trends) will be captured by *β*_2_ + *β*_3_. We will conduct tests with these null and alternative hypotheses: *H*_0_ : *β*_2_ + *β*_3_ = 0 vs. *H*_*A*_ : *β*_2_ + *β*_3_ ≠ 0.

### Secondary outcomes at practice level: Fidelity to EHR intervention

At the time of enrollment, each site that agreed to a scheduled visit was asked to complete three surveys prior to the launch of the EHR intervention. The first, a *practice needs assessment survey*, was administered either on paper or online via a web-enabled instrument developed in SNAP Survey software [55]. The survey assessed: (1) the estimated volume of patients seen per week at the site and the estimated proportion of patients that identify as South Asian; (2) provider knowledge and practices regarding hypertension control and the US Million Hearts initiative; (3) type of EHR system in use at the site and sophistication of its current usage; and (4) whether the site was interested in the CHW component, EHR component, or both components of the intervention. In the second survey, each provider within each site was asked to complete a *provider needs assessment to determine*: (1) basic demographics of each provider; (2) if/how each provider uses a standardized hypertension treatment protocol, clinical decision support software, the health registry, team-based management of care, and/or patient education/support groups; and (3) how the provider learns of hypertension guidelines. And third, the staff member most experienced with the EHR was asked to complete an *EHR Checklist survey*, which assessed: (1) MU and PCMH recognition status; (2) frequency of use of specific tools within the EHR, like customized templates, registry reports, and alerts, which allowed for an assessment of fidelity to key intervention components prior to the start of the EHR component of the intervention; and (3) where and how the practice documents counseling and education provided to patients. Throughout the EHR intervention, fidelity to intervention is assessed by collecting the type, frequency, and utilization of registry reports, alerts, and order sets for each practice.

### Primary study outcome at individual level: Individual-level BP control

For assessment of the CHW intervention, the primary outcome of interest is hypertension control after 6 months among patients receiving the CHW intervention compared to those who are not receiving the CHW intervention. For this analysis, a mixed effect logistic regression model will be used to estimate the CHW effect on hypertension, accounting for clustering by clinical site (see eq. 2):

2 $$ \log \left[\frac{P\left({Y}_{ij}=1\right)}{1-P\left({Y}_{ij}=1\right)}\right]=\mu +{\beta}_1{H}_i+{b}_j, $$

where *Y*_*ij*_ indicates state of hypertension control at the end of 6 months for patient *i* in site *j*. *Y*_*ij*_ = 1 if patient achieved hypertension control, *Y*_*ij*_ = 0 otherwise. *H*_*i*_ = 1 if patient *i* was randomized to CHW in the first 6 month period, *H*_*i*_ = 0 otherwise. *b*_*j*_ is a random effect for site *j* with mean 0 and variance ${\upsigma}_b^2$. *μ* + *b*_*i*_ is the log odds of hypertension control for patients randomized to the control group in the first 6 months. *β*_1_ represents the log odds ratio of hypertension control for those randomized to CHW compared to those randomized to the control group. We will conduct a test with the following null and alternative hypotheses: *H*_0_ : *β*_1_ = 0 vs. *H*_*A*_ : *β*_1_ ≠ 0 to assess whether or not the CHW intervention had an effect on hypertension control after 6 months.

A secondary analysis of individual-level data will also be conducted among HF patients with a diagnosis of hypertension to further assess factors that might be associated with improved outcomes following the implementation of the EHR intervention. The outcome for individual-level analyses is hypertension control at last visit (systolic blood pressure (SBP) <140 and diastolic blood pressure (DBP) <90) [56]. Through the partnership with HF, we collect body mass index, age, gender, HbA1c, cholesterol levels, zip code, smoking status and frequency, diabetes diagnosis, past cardiac events, and healthcare utilization information on individual HF patients seen in the office and had a diagnosis of hypertension in the year prior to the start of the EHR system at the practice. The analysis of the binary outcome (hypertension control) will be conducted using mixed effects logistic regression.

## Discussion

Project IMPACT builds upon the evidence base of the effectiveness of CHW and Million Hearts® Initiatives and proposes a unique integration of provider-based EHR and community-based CHW interventions. Millions Hearts® initiatives have demonstrated effectiveness at the health systems level and at the community level with English speaking minority communities [57, 58]. However, there exists a critical need to tailor, translate, and disseminate these initiatives for minority communities with limited English proficiency (LEP) with a high burden of CVD, such as the South Asian population. Thus, the focus of the IMPACT Project is the implementation and integration of evidence-based initiatives for the South Asian community to address hypertension control. Specifically, the study protocol described here tests the effectiveness of the CHW model embedded within the health care system to facilitate improved health outcomes by providing culturally tailored health promotion strategies, complementing physician-led efforts. Study findings can potentially provide translatable and scalable models for other LEP communities.

Despite its innovation, there are limitations that remain. The first, practices are not randomized to time-point. In other words, practices that enrolled first were the first to implement the EHR intervention, and subsequent practices that enrolled later receive the intervention at a later time. Randomization is not feasible for two reasons: (1) practice staff do not want to wait longer than is necessary to participate, and (2) recruitment and enrollment into the intervention is an intensive effort on the part of all partners involved, and waiting until 16 practices are enrolled would greatly delay study implementation and disrupt the study timeline. Second, our analysis of the impact of the EHR-component of the intervention is limited to HF participants only, given the nature of the partnership with HF and clinical sites.

This study’s strengths and innovation rests in several areas. First, the intervention takes evidence-based HIT strategies supported by the Million Hearts® Initiative and translates them to address CVD prevention among South Asians, a targeted disparity group with a high CVD burden and unique cultural barriers to health promotion and disease prevention. Second, the study integrates culturally tailored CHW programs into clinical practice initiatives to improve CVD outcomes for ethnic minority and immigrant populations with substantial language barriers to health care access and disease prevention. Using a hybrid effectiveness- implementation model, we evaluate the effectiveness of integrating these two evidence-based strategies (EHR-based tools for improved patient panel management and team-based care including CHWs) into health care delivery systems by partnering with a multi-stakeholder network that includes insurers, providers, and front-line quality improvement agencies. The partnerships guiding this protocol were critical to the process of provider engagement, accessing patient data to evaluate study effectiveness, and the provision of technical assistance and training. Cross-sector partnerships can enhance the sustainability of community-clinical linkage programs, and future studies should assess the potential and impact of partnering with multiple payers to enhance the reach of these types of programs and strategies. These findings will have implications for translating similar strategies for other LEP communities, including Asian and Hispanic Americans, and to other clinical settings. Finally, the project informs the effectiveness of these interventions in team-based care approaches, thereby, helping to develop relevant sustainability strategies.

## Abbreviations

BP: Blood pressure
CHW: Community health worker
CMS: Centers for Medicare & Medicaid Services
CVD: Cardiovascular disease
eCW: eClinicalWorks
EHR: Electronic health record
HF: Healthfirst
HIT: Health Information Technology
IMPACT: Improving Millions Hearts for Provider and Community Transformation
LEP: limited English proficiency
MOU: Memorandum of Understanding
MU: Meaningful Use
NCQA: National Committee for Quality Assurance
NHLBI: National Heart, Lung, and Blood Institute
NYC HANES: NYC Health and Nutrition Examination Survey
NYC: New York City
NYU-CUNY PRC: New York University-City University of New York Prevention Research Center
PCMH: Patient-Centered Medical Home
PCP: Primary care provider
